# Organizational e-Health Readiness: How to Prepare the Primary Healthcare Providers’ Services for Digital Transformation

**DOI:** 10.3390/ijerph19073973

**Published:** 2022-03-27

**Authors:** Agnieszka Kruszyńska-Fischbach, Sylwia Sysko-Romańczuk, Tomasz M. Napiórkowski, Anna Napiórkowska, Dariusz Kozakiewicz

**Affiliations:** 1 Faculty of Management, Warsaw University of Technology, 02-524 Warsaw, Poland; dariusz.kozakiewicz.dokt@pw.edu.pl; 2 Institute of World Economy, Warsaw School of Economics, 02-554 Warsaw, Poland; tnapio@sgh.waw.pl (T.M.N.); atalip@sgh.waw.pl (A.N.)

**Keywords:** COVID-19, e-health, innovation in health and care, technology enabled care, organizational readiness, PLS-SEM method, digital transformation, primary healthcare providers’ services

## Abstract

The COVID-19 pandemic has had two main consequences for the organization of treatment in primary healthcare: restricted patients’ access to medical facilities and limited social mobility. In turn, these consequences pose a great challenge for patients and healthcare providers, i.e., the limited personal contact with medical professionals. This can be eased by new digital technology. While providing solutions to many problems, this technology poses several organizational challenges for healthcare system participants. As the current global situation and the outbreak of the humanitarian crisis in Europe show, these and other likely emergencies amplify the need to learn the lessons and prepare organizations for exceptional rapid changes. Therefore, a question arises of whether organizations are ready to use modern e-health solutions in the context of a rapidly and radically changing situation, and how this readiness can be verified. The aim of this article is to clarify the organizational e-heath readiness concept of Polish primary healthcare units. This study employs the triangulation of analytical methods, as it uses: (i) a literature review of e-health readiness assessment, (ii) primary data obtained with a survey (random sampling of 371 managers of PHC facilities across Poland) and (iii) the Partial Least Squares Structural Equation Modeling (PLS-SEM) method, employed to estimate the structural model. The evaluation of the model revealed that its concept was adequate for more mature entities that focus on the patient- and employee-oriented purpose of digitization, and on assuring excellent experience derived from a consistent care process. In the context of patients’ restricted access to medical facilities and limited social mobility, a simpler version of the research model assesses the readiness more adequately. Finally, the study increases the knowledge base of assets (resources and capabilities), which will help healthcare systems better understand the challenges surrounding the adoption and scaling of e-health technologies.

## 1. Introduction

The complexity of the healthcare system has been consistently increasing—as noted in a quote from the eminent pediatrician Sir Cyril Chantler, published in the British Medical Journal (BMJ) in 1998 [1]: “Medicine used to be simple, ineffective, and relatively safe. Now it is complex, effective and potentially dangerous”. The COVID-19 pandemic, announced by the World Health Organization in March 2020, has added to this complexity by creating the need for an urgent digital transformation to ensure the continuity of patient primary care.

Primary care physicians play a vital role in the health of individuals, families, and communities. They serve as the first point of contact and as dedicated, longitudinal care advocates; they also have an ongoing responsibility to their patients’ comprehensive care needs—chronic, preventive, and acute—across care settings.

Personal contact with medical professionals has turned out to be one of the greatest challenges during the COVID-19-induced conditions that have restricted patients’ access to medical facilities and limited social mobility. Current Information and Communication Technology (ICT) in the field of telemedicine and the daily use of mobile applications by patients proved helpful with this issue [2]. The described situation also sheds new light on the lack of readiness of primary healthcare (PHC) organizations to use technological solutions, which had to be implemented within days, and not months or years [3,4]. Conventional health services have been replaced by remote and ICT technology-powered medical services aiming to ensure continuity of care processes [5]. Furthermore, the acceleration of recent trends in how consumers seek out care have yielded solutions, which more seamlessly and conveniently integrate aspects of primary care into patients’ everyday lives. The social distancing guidelines implemented during the COVID-19 pandemic have fueled this shift further—toward digital and virtual care solutions in particular [6].

In principle, ICT contributes to solving many problems, and creates new opportunities to challenges faced by traditional healthcare [7,8,9,10]. ICT has been recognized as an essential tool in enhancing healthcare quality, accessibility, and delivery [11,12,13]. It is currently challenging to imagine a healthcare entity not using some form of IT solutions. This process of integrating IT solutions into healthcare is expected to accelerate [14]. Three challenges have been recognized as factors increasing the pressure for a call to action in the healthcare digital transformation process: (1) organizational transformation [15,16], (2) process innovation [17,18] and (3) process-oriented technology [17,19,20].

Digital transformation is not a transition, but a radical change, which is associated with the simultaneous implementation of activities in the three above-mentioned areas [21]. While the second and third topics are derived from the improvement of operational processes, the first concerns strategic orientation.

As geographically diverse research studies show, primary healthcare organizational transformation depends on organizational readiness and requires an appropriate analytical approach to the digitization effort [22,23,24]. An effective degree of organizational readiness for digital transformation (organizational e-health readiness, OeHR) assessment is also needed to avert failures and ensure trust while increasing primary care system benefits. Technology acceptance and successful adoption comes with trust, which accelerates its implementation [25,26]. There are numerous factors contributing to the prevailing levels of trust and readiness of health professionals as regards implementing e-health [27]. Therefore, a model to analyze PHSs’ digital status quo is needed to support these organizations in defining their digital transformation roadmap. Because of this, research on OeHR [28], resulting in scientifically designed models aimed at assessing the organizational e-health readiness of PHC facilities, is required.

The aim of this paper is to (i) clarify the organizational e-heath readiness of Polish PHC facilities and (ii) design and evaluate the constructed research model. The OeHR model is built on the definition from [29], which defines such a model as ‘‘an abstract representation of objects and events from the real world for the purpose of simulating a process, predicting an outcome, or characterizing a phenomenon’’. These three purposes may be viewed as describing, predicting and estimating. In this paper, we are mostly interested in the former, i.e., a model derived from a literature review and evaluated through hypotheses testing. This work is the result of a compromise between striving to describe the phenomenon as closely as possible, and achieving its maximum simplification for operational purposes.

There is no universally applicable approach to model evaluation [30,31,32,33,34]. We follow Beck et al. [32], who refer to two aspects of a model’s evaluation: composition and performance. The composition of the model refers to the way the constituent hypotheses are formulated and assembled. Performance refers to the acceptability and usefulness of the model’s outputs for the intended task. While composition is an internal measure of the model’s reliability, performance is an external measure. These two aspects of model evaluation are echoed repeatedly in related literature, and each has its own logical means of assessment.

This paper employs a triangulation of analytical methods to test its research hypothesis, which concern the statistically significant effects of (i) the digital strategy on building digital competences, (ii) the digital strategy on building an organizational culture conducive to digital transformation, (iii) building digital competences on the development of technology, (iv) an organizational culture conducive to digital transformation on the development of technology, (v) an organizational structure conducive to transformation on the development of technology and (vi) the organizational culture on the structural readiness of an organization favoring the digital transformation in PHC units. Firstly, a literature review of e-health readiness assessments was carried out to provide background knowledge for both the research model conceptualization and hypotheses formulation. Secondly, primary data were obtained via a survey (random sampling of 371 managers of PHC facilities across Poland). Thirdly, a Partial Least Squares Structural Equation Modeling (PLS-SEM) method was used to estimate the structural model aimed at testing our research hypothesis [35].

Our study contributes to the understanding of the organizational e-health readiness dimensions of primary healthcare, and how they can be integrated into a synthetic model. Specifically, the contributions of this work include a systematic bibliographic review that led to the conceptualization of an organizational e-health readiness assessment model. The authors used the model along with primary data obtained from a survey carried out among 371 managers of healthcare institutions all over Poland. Furthermore, a multivariate analytical model (PLS-SEM) was used to statistically establish relationships between the studied variables, and therefore evaluate the final model. The research findings can help healthcare actors gain a holistic view of the relationships between the model’s dimensions and their impacts on digital transformation e-readiness. Finally, the study increases the current knowledge base of assets (resources and capabilities), which will help healthcare systems better understand the challenges surrounding the adoption and scaling of e-health technologies. This research is a part of an ongoing study (2020–2022) aiming to examine the organizational e-health readiness of primary healthcare for digital transformation in Poland, including the implementation of modern telemedicine tools during the COVID-19 pandemic.

This paper is structured as follows. Section 2 presents a literature review on the dimensions of e-health readiness, resulting in the development of the OeHR research model. Section 3 outlines the specifications of the applied research methods, including a presentation of the constructs and variables used and an explanation of the data gathering process. Section 4 gives the results obtained via the study of the factors in the model of organizational e-health readiness for digital transformation. Section 5 consists of a discussion of the empirical consequences of the literature-derived OeHR model. Finally, the paper also provides conclusions and practical implications.

## 2. Literature Review

### 2.1. Dimensions of e-Health Readiness—Literature Reiview

A range of frameworks and dimensions are used in assessing e-health readiness, and these are described in the literature [36,37]. The body of knowledge on e-health readiness is dispersed and inconsistent, while some dimensions overlap with others. However, one or more of the following readiness dimensions/determinants were present in most studies dealing with the concept of e-health readiness: core/need, engagement, technology, society, learning, policy, and user acceptance and use. Table 1 presents the results of the conducted literature analysis.

#### 2.1.1. Core/Need Readiness

Core or need readiness is the most frequently used dimension in the assessment of e-health readiness. It appeared in all of the reviewed studies. However, the individual meanings and interpretations of this determinant differed slightly, as various authors approached this dimension differently. In defining core readiness, most authors focused on the realization of needs or problems, and expressed dissatisfaction with the present situation and conditions [28,39,42,46,47]. When the realized problems were more serious and the dissatisfaction expressed by physicians was higher, healthcare organizations and providers presented a greater degree of readiness to adopt new practices to create change. Campbell et al. used the term efficacy, which refers to participants’ desire to determine whether e-health solutions would fill a functional need in their practice before they invested time and money in making a change [40]. Staff attitudes and perceptions of the potential use of the technology were also mentioned as an important factor by Demiris et al. [41]. Overhage et al. assessed the goals and functionality, verifying what problems the solution would try to solve, as well as plans for sustainable business models [44]. Khoja et al. [38] defined this category by putting more emphasis on the overall planning process for a proposed e-health program, as well as the knowledge and experience of planners with programs using ICT. In addition to needs assessment, specific core readiness items included the key aspects of planning, as well as the determinants of accessibility, such as the appropriateness of the technology and the integration of the technology with existing services. Similarly, Jennett et al. referred to planning readiness, which included a telemedicine strategic plan, needs assessment and analysis, and leadership readiness [24]. Kgasi et al. [48] added to the above-mentioned factors (i.e., need for change, dissatisfaction with manual systems, self-efficacy, e-health project planning, trust in the use of technology) another two: awareness and willingness to change. A different approach was presented by Lennon et al. [45], as they assessed the readiness on macro, meso and micro levels. On the meso level, they covered discontinuity and organizational culture, and they discussed the development of the organizations’ digital strategies and/or the adoption of e-health solutions. Lennon et al. [45] concluded that to be successful, a digital health innovation must be closely aligned with the health service’s organizational vision and road maps for change.

#### 2.1.2. Engagement Readiness

A second e-health readiness determinant identified in the literature has been defined as engagement readiness. Engagement involves exposure to new solutions and the active participation of people in the idea of e-health. In this process, people recognize and weigh the perceived advantages and potential disadvantages of e-health, assess risks, and actively question the chosen solution. Engagement allows people to express their hopes, fears and concerns about adopting new technology, providing insight into the factors that potentially facilitate or impede the further development of e-health readiness [28,39,42,43,46,47]. Given their sense of curiosity, people critically enquire to determine the cost–benefit balance of e-Health adoption, both immediate and long-term [46,47].

Readiness among individuals in an organization can vary from absolute refusal to cautionary interest [42]. Therefore, it is reasonable for it to include the factors suggested by Parker Oliver et al.: assessing personal use of technology by employees, and employees’ comfort with technology [41]. Similarly, Lennon et al. defined micro-level readiness, which embraces (among other things) health professional readiness (workload and professional confidence), the agency of individuals and their perceptions of “consumer” digital health tools, and trust in “consumer-facing” digital technologies. Workload pressures and the lack of capacity were recurring barriers to incorporating new technologies into everyday working practices. Occasionally, incentives had to be used to overcome this challenge. Concerns about data security when partnering with private companies were also a significant barrier. The requirement of data entry and/or a change in daily routines affected the readiness of users to adopt these technologies. Trust in digital health security was an issue related to concerns about the safety and security of privately held data, and whether it would be shared with organizations without consent [45]. According to Coleman et al. and the analogy of Activity Theory, doctors’ and hospital administrators’ willingness to make initial investments clearly show that they are ready to engage with the idea of e-health [43].

Engagement readiness includes demonstrations of leadership. Research has shown that constant change has made ownership and responsibility for digital health services unclear, and a lack of senior management buy-in was identified as a barrier to organizational readiness for digital health, negatively affecting implementation efforts [44,45]. A specific group of leaders who were enthusiastic promoters of innovation were called innovators or champions. Innovators, whether they were genuinely interested in the innovation or frustrated with the current situation, provided an example of working with technology that could encourage and motivate others [42]. Clinical endorsement could involve a single health professional (a social opinion leader) endorsing it to people in their practice, or (more likely) a body of clinicians publicly stating that a product or a solution was useful and clinically beneficial [45]. Champions are often thought to be essential to the successful implementation of new solutions [42].

The activity of innovators accelerated the learning process in organizations through intragroup dynamics [42]. Li et al. [39] described engagement as healthcare providers’ exposure to potential new solutions and their willingness to accept the training needed to assimilate technology.

Rezai-Rad et al. included factors from e-health education to engagement readiness [28]. Campbell found three themes related to engagement: care providers’ perception of telemedicine as a threat to their livelihood, professional autonomy, or both (Turf); the apprehension of individual providers (Apprehension); professional and emotional involvement in the technology, stakeholders’ acknowledgement of its benefits, adaption to their needs, and offering of assistance to others learning the solution (Ownership) [40].

Engagement, understood as support in the implementation of a new solution, was described by Jennett et al. [24]. The authors used the overarching terms workplace and human resource readiness, which embrace the telemedicine coordinator, change management readiness, and roles and responsibilities. Facilitation and support were used as factors in the e-health readiness model by Kgasi et al. [48]. The authors also referred to the preparedness of the nation in terms of government commitment and the legal infrastructure available to promote, support, sponsor, facilitate and regulate e-health, and its various requirements. The building of efficient structures to support the implementation of e-health was the main element of structural readiness—the overarching term embracing attributes related to institutional and human resource structures [46,47].

#### 2.1.3. Technological Readiness

Technological readiness addresses the availability and affordability of the required ICT, as well as the hardware and software needed to implement a proposed solution [38]. It also aims to determine the use of existing ICT infrastructure (hardware) and available electronic resources (software), as well as IT support personnel and healthcare providers’ past IT experience [39,41,42,43]. Some authors specify additional technical categories and their characteristics, such as network [43], data standards, replicable and scalable tools [44], infrastructure quality and IT security [28]. Jennett et al. summarized these factors as part of workplace readiness, which should help deliver efficient and appropriate technology and assure adequate access [24].

Physical access to technology, along with determinants of accessibility such as affordability and capacity building, was identified as an important component of technological readiness [28,38,42]. Included within the definition of access were: (i) appropriate scheduling (so that patients and practitioners can access equipment when needed), (ii) appropriate space (for utilizing the equipment and making repairs) and (iii) access to nonclinical applications (e.g., Web-based health information) [42].

Some authors [24,47,48] used the overarching term structural readiness, which is related to the perception of the soundness and preparedness of the e-health structure. Its attributes include technical resources, such as speed, the quality and reliability of the network, hardware and software compatibility, the availability of the ICT support team, as well as the availability and accessibility of the internet.

Lennon et al. [45] referred to technological readiness on the macro, meso and micro levels. Interoperability and infrastructure played a key role on the macro level. When talking about the market of solutions, interoperability was a key issue for digital health products and services. Organizations at the local and national levels need to invest in information technology infrastructures if digital services are to be rolled out and supported. Meso-level readiness also refers to the Information Technology Infrastructure. Variations in workflow processes and in-house IT and data management systems impede the advancement and integration of digital health initiatives. Technological readiness is an issue at the local organizational level, and refers to legacy systems, firewalls and strict information security procedures, which vary from site to site and leave health professionals and implementers ill-equipped to readily deploy solutions implemented elsewhere. Micro-level readiness refers to access to digital resources. Even when staff are digitally literate, it is impossible to drive new digital health services forward if technical infrastructure issues occur. For example, the poor connectivity of mobile devices such as tablet computers, and firewalls that block access to the internet, prevent engagement with new digital health applications and services.

In technological readiness, it is important that a certain degree of experimentation is permitted when using new technologies, and organizations need to encourage practitioners to be creative in their use of equipment [42].

#### 2.1.4. Societal Readiness

Societal readiness refers to the level of preparedness of a health institution and its staff to participate in the networked world. It refers to the attempt to understand the existing interactions and communication links of the concerned institution with other healthcare institutions, determinants of accessibility such as relevance, and the provision of care in collaboration with other healthcare organizations [24,28,38,39,43,48]. Specific societal readiness items deal with sociocultural factors, and may address the issues of inequity in gender and social classes [38]. Further, they involve internal communication among healthcare providers, which depends on communication mediums and frequency [39]. Rezai-Rad et al. [28] noted the importance of trust, which is of high importance but is absent in other models.

Societal readiness is defined by some authors as practice context, which is of special importance for rural areas, where technological change moves at a slower pace than in urban communities [40]. The relationship between individuals and their environment was considered through the component of community [43] and demonstrations of community commitment [44].

Some authors [24,42] used the overarching term of structural readiness, which included communication and participation. Open communication during the planning and implementation phases was critical in achieving support for e-health programs, as well as in facilitating change and team building

Health organization played a role in influencing other sectors to adopt e-health innovations. Inter-sector cooperation could help alleviate fears, address concerns, communicate ideas and mediate tensions among various groups. Hence, it was crucial in ensuring successful implementation [42]. Furthermore, Lennon et al. [45] raised the issue of industry readiness. Digital health is constantly promoted as a potentially lucrative market. However, enticing commercial entities to invest in opportunities in emerging digital health, wellbeing or social care sectors is not as straightforward as originally anticipated.

Lennon et al. [45] emphasized relevance of codesign in meso-level readiness. Codesign methodologies and intensive consumer engagement (if utilized successfully) can resolve knowledge gaps relating to consumer preferences.

#### 2.1.5. Learning Readiness

Certain indications of factors related to learning are visible in the dimension of engagement. Their significance, as emphasized by many authors, allowed us to distinguish a category related to competences and learning readiness.

Clinicians and healthcare providers may fear e-health, as they believe it could be used to disempower and, in some cases, replace them. However, the main barrier was not the fear of role replacement, but a lack of knowledge and skills related to e-health, which significantly affected how they engaged with new technologies. The pace of technological change made clinicians feel unable to keep up to date with new and emerging developments. Hence, training, and the alignment with professional roles and identities, should be assessed when determining learning readiness [45].

Preparing staff to adopt e-health as part of their workplace has been identified as a component of an organization’s structural readiness [24,42,48]. This category concerns issues related to the existence of programs and resources providing training to healthcare providers in using the technology [38,42,48]. These include an orientation (introduction) session, procedural resources, knowledgeable supervisory staff, and “hands-on” training [42]. Specific learning readiness items deal with the inclusion of healthcare providers in the planning process, and determinants of accessibility such as capacity building [38].

Campbell underlined the importance of time to learn, which refers to the hesitancy among providers in taking the time to learn a new technology and persuading patients of its worth [40].

#### 2.1.6. Policy Readiness

According to Jennett et al., written policies are a means to build the structural readiness of organizations (and hence workplace readiness) for the adoption of e-health. While the necessity of appropriate policies is obvious, it is not clear who should be responsible for creating and maintaining these policies [24,42].

Khoja et al. defines policy readiness at institutional and government levels. Policies at the government and institutional levels address common issues such as licensing, liability and reimbursement. Specific policy readiness items include determinants of accessibilitys such as the legal and regulatory framework, and political will [38]. Kgasi et al. put even more emphasis on the role of government readiness, referring to the preparedness of the nation state in terms of government commitment and the legal infrastructure to promote, support, sponsor, facilitate and regulate e-health, as well as its various requirements [48]. Political readiness and national policy—in terms of information governance policies and legislation issues within the health and social care sectors—were brought up by Lennon et al. The authors emphasize the importance of policy readiness in terms of risk and liability. Furthermore, the accreditation and official endorsement of e-health products and services likely influence deployments and the future development of e-health, while further research and policy work is required to clarify what apps and services require accreditation, and what such accreditation should look like [45].

#### 2.1.7. Acceptance and Use Readiness

Acceptance and use readiness refers to the effort and performance expectancy, and therefore, the intention to accept and use e-health technology [46,47,48]. The effort expectance may include personal factors such as age, academic qualifications, experience with networking technology, ability to use the technology, and the technology’s newness. On the other hand, performance expectance entails those attributes that lead to satisfaction with technology, organizational awareness and expected benefits [48]. Additionally, this category may be measured by indicators such as attitudes toward using ICT in healthcare management, perceptions of the usefulness of ICT to job performance, perceived ease of use, social influence and facilitation, and conditions for using ICT [47].

Variations in digital literacy and access may cause widespread problems while implementing e-health solutions. Research has shown that on both sides (the healthcare provider and the patient), users range from younger consumers—who were more adept, confident and ready to use digital tools—to older adults with little or no previous awareness or understanding of basic IT. Despite reported the growing use of digital tablets and smartphones among the general population, many people still lack basic access to mobile devices. The cost of technology, and poor access to computer equipment and free internet services, prevent many individuals from participating in e-health solutions [45].

The resource-driven dimensions of organizational readiness are grounded in the resource-based view (RBV) of the organization, which assumes resource heterogeneity and resource immobility across organizations. In this framework, an organization is a bundle of resources, capabilities or routines, which create value and cannot be easily imitated or appropriated by competitors due to isolating mechanisms. Initially developed through a series of papers by several authors in the 1980s–1990s, major extensions and refinements of the RBV include the knowledge-based view (KBV) of the firm, dynamic capabilities, and the relational view, which recognize that capabilities can be developed and shared through alliances between firms [49].

### 2.2. Conceptualization of the OeHR Research Model

The COVID-19 pandemic forced an inevitable change in healthcare systems around the world. The healthcare delivery process had to be adjusted right away, without any delays, to assure the continuous efficacy and performance of the system. This placed a different emphasis on the readiness assessment models developed so far. Rapid and radical changes require a more flexible approach [50,51]; hence, the OeHR research model (grounded in dimensions derived from RBV) was further developed on dimensions grounded in the concept of dynamic capabilities [52]. A dynamic capability is the PHC facility’s ability to integrate, build and reconfigure internal and external competences [53] in order to build, integrate, or reconfigure its operational capabilities [54] in environments of rapid technological change [53].

**Figure 1:**
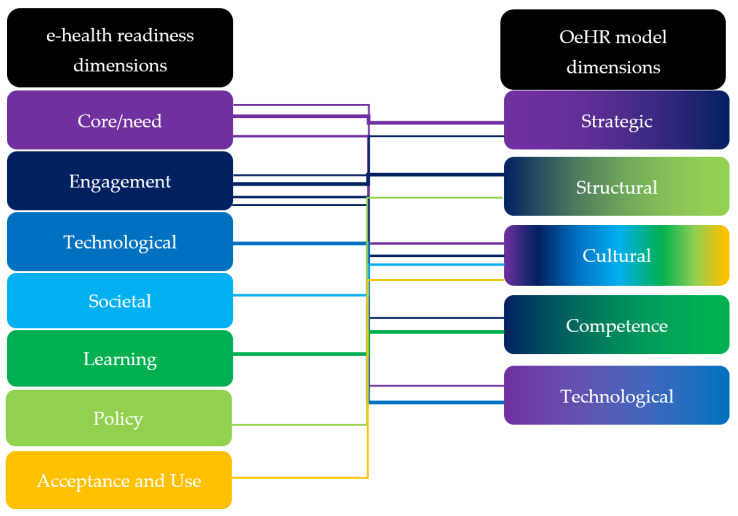
Conceptual process of developing the dimensions of the OeHR research model.

Figure 1 presents the conceptual process of the OeHR research model’s development, based on the conducted literature review. The construction of the dimensions of the model has been supported by analyses of the literature on subdimensions, which are the constituent elements that make up a dimension.

#### 2.2.1. Strategic e-Health Readiness (STeHR) Dimension

There are numerous barriers that have been identified in the process of e-health solution implementation. Evidence shows that one of these is a lack of prior planning, which existing literature notes as essential. The lack of e-health strategy remains one of the major barriers that hinders efforts to implement e-health solutions [55]. A tailor-made, needs-based intervention strategy could assist organizations in adopting new technologies appropriately [56].

Strategic e-health readiness refers to the expression of digital solution implementation intent in the development and strategic goals of the organization [24,38,44,45,48,57,58], along with strong support from executives and leaders in its realization [24,45,48,57,58], which is important in changing the healthcare delivery process by the usage of new technologies [38,48,57,58]. This is done to assure excellent patient experiences, which are at the core of each digital transformation [58,59].

Hence, there are four literature-related subdimensions—a customization option that isolates a hierarchy of categories from the rest of the dimensions that define the strategic e-health readiness: strategic direction, innovativeness, leadership support, and focus on patients’ experience.

#### 2.2.2. Competence e-Health Readiness (CMeHR) Dimension

Organizations with ICT-based or digital resources, competences, and knowledge are more likely to move from information system applications working in silos, to high frontier digital technologies such as data analytics, artificial intelligence (AI), internet of things (IoT) and social media [60]. Technological progress requires skills upgrades through the explicit learning of new technologies. An organization’s performance is highly associated with its learning capabilities, levels of technology, and a host of firm-level knowledge, skills and experiences. Some studies have shown that ICTs (particularly in the healthcare sector) remain underused by healthcare professionals as they lack knowledge on the best strategies to integrate them into their practice [61].

Competent e-health readiness addresses issues related to the existence of tools and methods dedicated to deploying patient-focused digital solutions [24], as well as to promoting a digitally supported way of working [24,28,38,39,41,42,43,46,47,48], providing resources for training on the use of the technology [28,38,42,45,48,57], and the availability of digitally competent leaders [40,42,44,45], experts [24,40,41,42,45,48,57] and employees [41,42,45,57].

Hence, there are five literature-related subdimensions defining competent e-health readiness: work methods and tools, leadership competences, education, experts’ allocation, and employees’ competences.

#### 2.2.3. Cultural e-Health Readiness (CLeHR) Dimension

An e-health-ready culture places the patient and the continuum of care process at the top of the priorities of the organization [45,58]. Their roles and responsibilities are clear, and they have adequate goals and metrics to assess the success of the undertaken implementations [24,45]. Research on patients’ satisfaction, the continuity of care process and operational effectiveness is being continuously conducted, and a feedback loop is assured in the design and development of digital solutions [45,46,47,48,57]. Experimentation and risk are accepted as regular practice [42]. Cooperation with partners is undertaken to create superior solutions for patients. All these cultural change processes are supported by strong internal and external communication [24,28,38,39,42,43,48,57].

Hence, there are six literature-related subdimensions defining cultural e-health readiness: operational results, patients’ experience, continuous learning, communication, openness to experiments and partnerships.

#### 2.2.4. Structural e-Health Readiness (SCeHR) Dimension

Structural readiness focuses on the establishment of efficient structures as a foundation for successful e-health projects within an organization [42].

Structural e-health readiness encompasses standardized processes and policies [24,42,45,57], with the primary focus being on the patient care process, the allocation of appropriate resources to work on digitization [24,38,42,46,47,48,57], and the establishment of structures enabling the networking of all professional groups within an organization to imagine, co-design and co-create new solutions [24,38,45,46,47,57].

Hence, there are five literature-related subdimensions defining structural e-health readiness: continuity of care processes, process standardization, resource allocation, internal networking, and co-creation and ideation.

#### 2.2.5. Technological e-Health Readiness (TCeHR) Dimension

Technological e-health readiness determines workplace readiness, which should help deliver efficient and appropriate technology and assure adequate access [24]. It encompasses agility in the way the work on digital solutions is organized [45,57], including flexible funding schemes [24,42,44,45], the use of a supportive digital workplace, including access to new technologies [24,28,38,39,42,43,45,46,47,57], and efficient everyday IT support [24,39,41,42,43,47,48].

Hence, there are four literature-related subdimensions defining technological e-health readiness: agility, IT architecture, usage of new technologies and continuity of process support.

## 3. Materials and Methods

### 3.1. Methodology for Research Model Evaluation

This paper employs a triangulation of analytical methods to evaluate the research model. In the first step, a literature review of e-health readiness assessment was carried out to provide background knowledge on both the research model’s conceptualization and hypothesis formulation for our OeHR model evaluation.

In this process, three key research questions we addressed:

1. What are the key dimensions that constitute and organize the e-health readiness model?
2. What relationships exist among the identified key dimensions?
3. How are the dimensions described and analyzed?

In the second step, primary data were obtained via a survey (random sampling) of 371 managers of PHC facilities across Poland. In the third step, a Partial Least Squares Structural Equation Modeling (PLS-SEM) method was used to estimate the structural model aimed at testing our research hypothesis [35]. Keeping in mind that a responses:questions ratio of 10:1 is best, 5:1 satisfactory and 3:1 acceptable [25], our 11.59:1 ratio is appropriate for the proposed empirical method.

### 3.2. Hypotheses Formulation

Following the outcomes of the systematic literature review, intended to conceptualize the research model, we developed three groups of hypotheses to evaluate our research model:

1. effect of strategic e-health readiness on cultural and competency dimensions;
2. effect of competency, and cultural and structural e-health readiness, on the technological dimension;
3. effect of cultural e-health readiness on the structural dimension.

For the health sector to tap into the benefits of e-health, organizations need to have the right strategies in place. However, the presence of a strategy to guide implementation is not sufficient. If the setting is not “ready” to use these innovations, their implementation and use will not happen, or will be inefficient [62]. When preparing the organization, a strategy may play a facilitating role, and should include goals concerning adequate training, knowledge, and skills development, as well as cultural transformation. A lack of knowledge and skills, and resistance from healthcare workers, hinder the adopting of e-health solutions. The employed strategies should address these issues so as to prepare the way for the implementation of technology [55]. An e-health implementation strategy based on learning from the past and avoiding mistakes that others have made will allow for an optimal decision-making process. Consultation with end users and sharing information in various ways can promote knowledge translation [56].

To verify the assumed impact of strategic e-health readiness on cultural and competency dimensions, the following hypotheses have been proposed:

**H1:**  *There is a statistically significant effect of the digital strategy on building digital competences in PHC units (regression path: STR → KOMP).*

**H2:**  *There is a statistically significant effect of the digital strategy on building an organizational culture conducive to digital transformation in PHC units (regression path: STR → KUL).*

To assure sustained technology implementation, first, the setting needs to be ready to use an innovation [62]. E-health readiness in relation to users’ and managers’ knowledge and skills, policies, regulations, and guidelines, as well as staff attitudes, should be assessed prior to implementing any e-health solution [55]. Sustainable technology implementation requires evidence and needs-based applications, a skilled and knowledgeable workforce, and a “ready” setting [55]. Specific patient, healthcare provider and organizational needs identification (KUL) allows us to overcome technical challenges [56]. Successful digital transformation projects have a well-established digital culture at their core, which can translate the capabilities of digital technologies to the knowledge capabilities needed for enhancing adaptability and flexibility, sustainable innovation adoption, and continuous improvements in wellbeing-related outcomes [60].

To verify the assumed effects of competence, and cultural and structural e-health readiness, on the technological dimension, the following hypotheses have been proposed:

**H3:**  *There is a statistically significant effect of building digital competences on the development of technology in PHC units (regression path: KOMP → TECH).*

**H4:**  *There is a statistically significant effect of the organizational culture conducive to digital transformation on the development of technology in PHC units (regression path: KUL → TECH).*

**H5:**  *There is a statistically significant effect of the organizational structure conducive to transformation on the development of technology in PHC units (regression path: ORG → TECH).*

Research by Yunis et al. [60] argues that digital transformation is never about a technology being implemented, but rather about a culture that needs to be nurtured to change behavior and drive result. An organizational culture is perceived as getting things done, or common characteristics of organizations shaping their members’ behaviors and enhancing (or impeding) their strategic achievement and performance. With its assumptions, values and norms, the culture influences the top management’s frame of reference, which shapes organizational structure.

To verify the assumed impact of cultural e-health readiness on the structural dimension, the following hypothesis has been proposed:

**H6:**  *There is a statistically significant effect of organizational culture on the structural readiness of an organization to favor the digital transformation in PHC units (regression path: KUL → ORG).*

### 3.3. Population and Data Collection

There are approximately 21,500 PHC facilities in Poland [63], from which 371 medical facilities were randomly selected, yielding a 5.04% estimation error, which constitutes a representative research sample [64]. Data were collected in August/September 2021 on behalf of the Warsaw University of Technology using the CATI method. The survey contained 32 questions divided into five dimensions: strategic, competence, cultural, structural, and technological, asking responders to agree or disagree with provided statements. Answers were presented using the Likert scale, coded as follows: 1—Strongly disagree, 2—Disagree somewhat, 3—Neither agree nor disagree, 4—Agree somewhat, 5—Strongly agree. All 32 statements were included in the analysis (Table 2). Managers were also asked several questions about the finances and staffing of the clinics under their responsibility. As those questions were voluntary, managers rarely provided responses.

### 3.4. Ethics

The questionnaire was prepared based on the literature review. The survey instrument was assessed by the research team of the Warsaw University of Technology. Additionally, the management of the PHC facility CortenMedic was consulted on our questionnaire. The content of the survey and rules for carrying it out were accepted by the Warsaw University of Technology Senate Committee for Professional Ethics. The survey was anonymous. Respondents completed the survey voluntarily and could withdraw from the process at any time. The questions were read to the managers of the PHC facilities by telephone. All responses were recorded in the database.

## 4. Results

**Figure 2:**
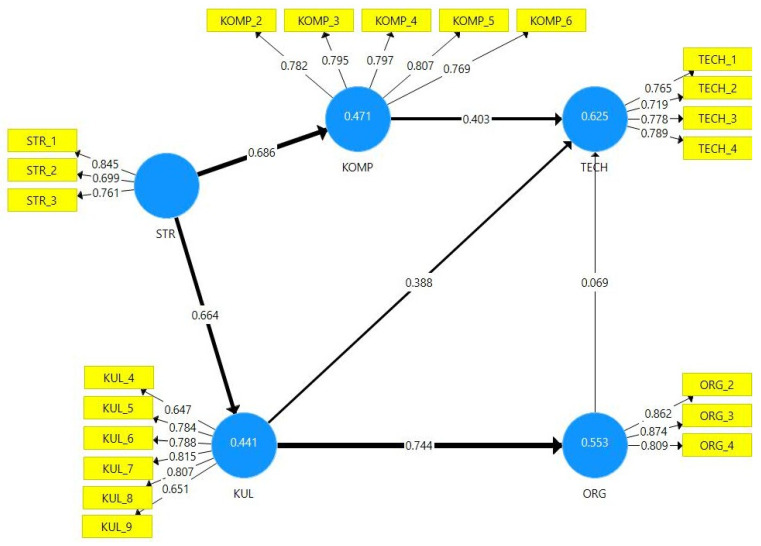
Structural model of dependencies between constructs (dimensions of the OeHR research model). *Figure description: (i) circles represent constructs; (ii) rectangles represent measurable variables (indicators); (iii) values given on the arrows between the indicators and the constructs represent factor loadings; (iv) values given on the arrows between constructs represent path coefficients (i.e., standardized regression coefficients); (v) values given inside the hidden constructs indicate the coefficients of determination R^2^.

The structural model (Figure 2) was estimated using the Partial Least Squares Structural Equation Modeling (PLS-SEM) method.

The evaluation of the model started with the measurement (external) part. For this purpose, indicators’ outer loadings were determined, and those with very low loadings, i.e., below 0.40 [65], as well as those with high levels of collinearity with other constructs, have been removed (STR_4, KOMP_1, KUL_1, KUL_2, KUL_3, KUL_10, ORG_1, ORG_5, ORG_6, ORG_7, TECH_5). Each removed element will be analyzed in detail in the Discussion section of this article. The reflective measurement model used in this study presents a relation from a hidden variable to its indicators [66]. In other words, it reflects the hidden properties of the measured construct.

Next, the model’s quality criterion Composite Reliability (CR) and its Average Variance Extracted (AVE) were analyzed. The minimum CR value in the PLS-SEM analysis should exceed 0.7 [67]. An AVE coefficient of 0.50 or more indicates that, on average, the construct explains more than half of the variance of its indicators [65]. In the presented model, all constructs present a CR value greater than the threshold level of 0.7 and an AVE value greater than the required minimum level of 50% (Table 3). This means that all reflective constructs of the model present a high level of convergent validity.

The Fornell–Larcker criterion was used to assess the discriminant validity [66]. The square roots of the AVEs of all model constructs, i.e., KOMP (0.790), KUL (0.721), ORG (0.849), STR (0.771), and TECH (0.763), are higher than the correlations of these constructs with other variables hidden in the model (Table 4). This means that the model meets another quality criterion, which is discriminant validity.

The next step in the analysis (i.e., the analysis of the structural—internal—model) was to test the set of research hypotheses. The first step was to examine the degree of collinearity. All measurable variables used in the model have a Variance Inflation Factor (VIF) index below 5.00 (Table 5), which indicates a lack of collinearity [66].

The most frequently used measure to evaluate the structural model is the coefficient of determination, i.e., R-squared (R^2^), calculated for each endogenous construct. The R^2^ value ranges from 0 to 1, with higher levels indicating higher levels of predictive accuracy [68]. The exogenous construct STR explains 47.1% of the variance of the endogenous construct KOMP (R^2^ = 0.471), and 44.1% of the variance of the construct KUL (R^2^ = 0.441). The variance of the endogenous construct TECH was explained at 62.5% (R^2^ = 0.625) by the remaining constructs of the model, i.e., STR, KOMP, KUL and ORG. The variance in the endogenous construct ORG was explained at 55.3% (R^2^ = 0.553) by the constructs STR and KUL.

Subsequently, the measurable variables were subjected to the bootstrapping procedure, i.e., testing the significance of the difference in path coefficients against zero (Table 6). The closer the path coefficients are to the value of 0, the weaker the relations [66].

The results of the structural model show that hypotheses H.1–H.4 and H.6 have been confirmed. The strongest relation was observed for KUL and ORG (path coefficient = 0.744, significance level < 0.001), followed by STR and KOMP (0.686, significance level <0.001), and STR and KUL (0.664, significance level < 0.001). A slightly weaker relationship can be seen in the case of KOMP and TECH (0.403, significance level < 0.001), as in KUL and TECH (0.388, significance level < 0.001). There is no significant relation between ORG and TECH (0.069, significance level = 0.279); therefore, hypothesis H.5 cannot be confirmed.

## 5. Discussion

The evaluation of the OeHR research model developed via the literature analysis of the phenomenon of organizational readiness for the digital transformation of PHC facilities was carried out via the verification of six hypotheses, the aim of which was to confirm the internal consistency between the dimensions of the developed model.

The researched relations have their empirical grounding in the classical science of management. Five out of the six hypotheses, describing the following relationships between five dimensions, have been confirmed (ordered according to the strength of the relationship): H6 (the effect of organizational culture on the organizational structure), H1 (the effect of organizational strategy on organizational competences), H2 (the effect of the organizational strategy on the organizational culture), H3 (the effect of organizational competences on technology) and H4 (effect of organizational culture on technology).

Only H5 (the effect of organizational structure on technology) was not confirmed. Structural e-health readiness and technological e-health readiness represent the PHC unit’s heterogeneous organizational and technical capabilities, which are related to design technologies, information and processes, and the acquisition and integration of external knowledge. In our study, the variables used to measure TCeHR are related to the ability of healthcare entities to integrate digital technologies, such as emerging technologies (e.g., voice interfaces, augmented reality, artificial intelligence, blockchain), patient experience tools and methods (such as persona and journey maps), and digital tools and modern architectures (APIs, cloud, etc.). We assumed that the technologies used in medical entities should be used to improve the patient care process, to design and modify digital solutions, to promote innovation, collaboration and mobility for doctors, medical staff and administrators, and to promote speed and flexibility in implementing digital solutions. To better understand the relationship between organizational and technological capabilities, we subjected it to a separate Exploratory Factor Analysis (EFA) and a Confirmatory Factor Analysis (CFA), which both confirmed it unequivocally. The results of the EFA and CFA analyses have been published in [4].

The verification of the hypotheses under study for PHC facilities enables the assertion that the evaluated model can also be used to analyze digital transformation efforts undertaken in other sectors of the healthcare system (e.g., outpatient care, specialist clinics, hospitals, clinical trials).

Moreover, based on both the literature review and our own empirical analysis, the definitions of the OeHR model dimensions were revised, and consequently the literature-related subdimensions in the research model were also modified.

In the consideration of strategic e-health readiness and competence e-health readiness, two statements from the model have been removed:

(1) The most important thing in my work is to ensure a good patient experience (variable STR_4);
(2) We use tools and methods related to patient experience, such as personas and travel maps, to design and modify digital solutions (variable KOMP_1).

In the context of the crisis (rapid change) caused by the unexpected pandemic of COVID-19, ensuring continuity in the care process itself was the focus of managers of PHC units, and in this particular situation, neither patient satisfaction nor the usage of digital-related tools to improve patient’s experiences were a priority [5,38,45]. Hence, the definitions of both dimensions were modified. However, the empirical results from the remaining statements show that 87% of PHC managers agreed or strongly agreed that the leaders support the implementation of digital solutions, which is in line with the results of [24,38,44,45,48]. Moreover, 81% of PHC managers agreed or strongly agreed that the implementation of digital solutions is an important element in PHC’s strategy, which is in line with [24,42,44,45,48]. PHC managers remained unsure (25%) when considering the usage of advanced technologies to change the way patient care is delivered. However, 57% confirmed that this is the case. Finally, the STeHR dimension is a composition of strategic direction, innovativeness, and leadership support. As regards competence e-health readiness, the strongest confirmation was given when assessing the development of employees’ digital competences (81%). The weakest assessed statement (which was still high) concerned the usage of digital tools to promote innovation, collaboration and mobility for physicians, healthcare professionals and administrations (67% agreed or strongly agreed, while 23% were uncertain). Finally, the CMeHR dimension is a combination of digital work tools, leadership competences, education, experts’ allocation, and employees’ competences.

In the case of cultural e-health readiness (CLeHR), four indicators have been removed:

(1) We have clear and measurable goals to measure the success of our digital solutions’ implementations (variable KUL_1);
(2) Each employee understands how their tasks are related to the effectiveness of digital solutions implementation (variable KUL_2);
(3) We have measures oriented towards patient satisfaction surveys (e.g., Net Promoter Score) (variable KUL_3);
(4) We work with partners and suppliers to create better solutions for our patients (variable KUL_10).

These indicators concern measures of the success of digital solution implementation, as well as of patient satisfaction, understanding of employees’ tasks’ impact on the effectiveness of digital solution implementation, and of cooperation with partners and suppliers. The PHC unit managers considered feedback from research, patients and operations; however, there are no measures used on a regular basis. Moreover, these managers are focused on the operations of their own units, and do not seek partnership. In the context of the crisis situation, PHC managers concentrate on simple solutions bringing immediate effects, and not on long-term solutions [39,43,62]. Empirical results show that at least 80% of PHC manager agree or strongly agree that they investigate how the channels of contact with the patient (e.g., visit, telecommunication) work together to ensure continuity in the patient care process, that conclusions from research and patient relations have a real impact on the selection and verification of digital solutions, and that they systematically draw conclusions from the operation of digital solutions and improve them. These results are in line with [38,46,47,48]. Finally, the CLeHR dimension is a combination of contact channel consistency, continuous learning, communication, and openness to experiments.

In the structural e-health readiness (SCeHR) dimension, four indicators have been removed:

(1) In our organization, the priority is the continuity of the patient care process, and not the individual tasks of individual employees (variable ORG_1);
(2) Medical, administrative, and technological employees jointly develop plans for the implementation of digital solutions (variable ORG_5);
(3) New ideas, solutions, or improvements in the organization of tele-consultancy came mainly from the management of the facility (variable ORG_6);
(4) New ideas, solutions, or improvements in the organization of tele-consultancy came mainly from the employees of the facility (variable ORG_7).

These indicators concern a shift in focus from individual tasks to the continuity of the process, as well as to the collaboration of all professional groups in planning digital solution implementation and gathering new ideas. The PHC managers agree or strongly agree that their organizational model encourages collaboration between doctors, medical and administrative staff and IT specialists (80%), that they dedicate appropriate resources to work on digitization (76%), and that there are defined, described and repeatable processes for the implementation of digital solutions (77%). Structural readiness focuses on the establishment of efficient structures as a foundation for successful e-health projects within an organization [42]. Finally, the SCeHR dimension is a combination of process standardization, resources allocation and internal networking.

In the technological e-health readiness (TCeHR) dimension, one indicator, concerning a shift of focus from the availability of IT systems to the continuity of the patient care process, has been removed:

When providing IT support, we focus on the continuity of the patient care process, not only on the availability of IT systems (variable TECH_5).

When the mobility of people and access to personal interaction is limited, and the only possible way to gain healthcare is through technology, PHC managers focus on access to IT systems supporting particular tasks and functions, and not necessarily on the continuity and consistency of the experience across the whole care process [24,38,39,42,43]. The empirical results show that PHC managers agree or strongly agree that they show a flexible, iterative, and collaborative approach to developing digital solutions (73%), and that the digital budget is flexible and allows them to change priorities (67%). The managers are less sure about the usage of modern technologies to improve the speed and flexibility of implementing digital solutions (58% agree or strongly agree, while 22% are uncertain) and to improve the patient care process (54% agree or strongly agree, while 24% remain uncertain). Finally, the TCeHR dimension is a combination of agility, IT architecture and the usage of new technologies.

The evaluation of the model reveals that its concept is suitable for more mature entities, which focus on the patient- and employee-oriented purpose of digitization, and on assuring their excellent experience as a result of a consistent care process. The digital transformation is thus only successful when continuity of the care process is assured. Continuity is the basis for trust in technology, which ensures reliable access to medical services [69,70]. This requires providing the option to systematically express ideas and co-create solutions based on them, with the usage of dedicated tools and methods (incl. personas, patients’ journey maps) and linking the digitization efforts to operational and patient-oriented results (incl. NPS). This linkage makes clear to the employees how their work contributes to the success of the implemented solutions. Mature organizations enter partnerships to build better solutions for their patients, and are more focused on the excellent care process than on working siloed IT systems. As the results of the research show, in the context of limited mobility and access to regular personal healthcare processes, the PHC managers focused on ensuring basic solutions, which enable them to continue performing basic tasks. Moreover, the bases of the solutions were given, and the PHC managers only had to focus on the execution, and not on ideation or co-creation.

This leads to the conclusion that the research model may be used to assess readiness in mature environments, and in contexts not limited by pandemic regulations. However, in a pandemic context, the evaluated research model assesses readiness more adequately.

## 6. Conclusions

In this study, we consider a dynamic capabilities-based approach to developing a new model of organizational e-health readiness. The organizational readiness of healthcare entities for digital transformation, as discussed in this article, is an essential aspect related to the inevitable need for remote medical care in the context of restrictions to patients’ access to medical facilities and limited social mobility. In this paper, we focus on the evaluation of a research model conceptualized based on a literature review.

In summary, the key findings from the model evaluation are:

(i) The conceptual OeHR research model developed based on the literature review may be used to assess readiness in digitally mature organizations, and in a context of open access to medical facilities and regular social mobility;
(ii) The evaluated OeHR research model is suitable to assessing the organizational readiness of PHC facilities in the context of limited social mobility and restricted personal access to healthcare providers;
(iii) Both the conceptual and the evaluated OeHR research models comprise five dimensions—strategical e-health readiness (STeHR), structural e-health readiness (SCeHR), cultural e-health readiness (CLeHR), competence e-health readiness (CMeHR), and technological e-health readiness (TCeHR);
(iv) The subdimensions that can be used to effectively measure the STeHR for the evaluated OeHR model were confirmed, and comprised strategic direction, innovativeness, and leadership support;
(v) The subdimensions that can effectively measure the SCeHR for the evaluated OeHR model were confirmed, and comprised processes standardization, resources allocation and internal networking;
(vi) The subdimensions that can effectively measure the CLeHR for the evaluated OeHR model were confirmed and comprised contact channel consistency, continuous learning, communication and openness to experiments;
(vii) The subdimensions that can effectively measure the CMeHR for the evaluated OeHR model were confirmed, and comprised digital work tools, leadership competences, education, experts’ allocation and employees’ competences;
(viii) The subdimensions that can effectively measure the TCeHR for the evaluated OeHR model were confirmed, and comprised agility, IT architecture and usage of new technologies;
(ix) The subdimensions that are not effective in assessing OeHR in the context of limited social mobility and restricted personal access to healthcare providers are focus on patients’ experience, continuity of care process, ideation and co-creation, operational and patient-oriented results, partnerships, methods and tools dedicated to patients’ experience, and continuity of process support.

The results of the study may, in a practical way, help managers of PHCs assess the readiness of their organizations for the digital transformation process in the context of rapid and radical changes, and address any identified barriers in this regard. Moreover, the results indicate the need to enhance the effort to ensure the continuity of care processes, which is the basis for developing trust in technology, and hence its successful adoption.

The promising results indicate the need for further research. The authors plan to identify the dominant models of organizational readiness for e-health among Polish POZ entities. In principle, each of the identified models will require a modification of the leadership concept and a change in professional and managerial competences, as well as the identification of technological needs, to ensure the continuity of the care process. In addition, it is important to incorporate patients’ perspectives, and the in-depth identification of barriers and drivers of organizational e-health readiness, in further research to guide public policy on the implementation of future e-health technologies.

## Figures and Tables

**Table 1:** e-Health readiness dimensions and their key attributes—literature review findings.

Dimension	Key Attributes	Sources
Core/need readiness	realization of needs or problems dissatisfaction with status quo expectations of potential solutions (efficacy) attitudes and perceptions of the potential use of technology plans of change knowledge and experience of planners appropriateness of technology leadership awareness and willingness to change digital strategy, goals, vision	[24,28,38,39,40,41,42,43,44,45,46,47,48]
Engagement readiness	awareness of the potential advantages and disadvantages of e-healthcare having a sense of curiosity or critical mindedness about the potential implications of e-healthcare adoption active questioning of e-healthcare as to what it could do and expressing hopes, fears, and concerns about adopting e-healthcare state of critical enquiry into the cost benefit analysis of e-healthcare adoption	[24,28,39,40,41,42,43,44,45,46,47,48]
Technological readiness	existing ICT infrastructure (hardware) available electronic resources (software) availability and affordability of required ICT IT support personnel healthcare providers’ past IT experience	[24,28,38,39,41,42,43,44,45,48]
Societal readiness	collaboration with other health institutions sharing of information provision of care to patients and communities in collaboration with other healthcare institutions sociocultural factors among staff (e.g., cultural factors; social roles and circumstance) socioeconomic position and sociocultural factors among clients and communities	[24,28,38,39,40,42,43,44,45,48]
Learning readiness	knowledge and skills in relation to e-health alignment with professional roles and identities existence of programs and resources to provide training inclusion of healthcare providers in the planning process accessibility of technology to learn time to learn	[24,38,40,42,45,48]
Policy readiness	existence of appropriate policies licensing, liability, and reimbursement government commitment and the legal infrastructure risk and liability accreditation and official endorsement	[24,42,44,45,48]
Acceptance and use readiness	experience with technology vendor support friendliness of use of e-health users’ satisfaction expected benefits services quality attitude toward using ICT in healthcare management perception of the usefulness of ICT in job performance perceived ease of use social influence and facilitation condition for using ICT	[46,47,48]

**Table 2:** Survey statements for the five dimensions of the OeHR research model.

Dimension	Variable Name	Statements
Strategic e-health readiness	STR_1	The implementation of digital solutions is an important element of our development (strategy) (variable STR_1);
	STR_2	We change the way we deliver patient care with technologies such as AI, API, and the internet of things (variable STR_2);
	STR_3	The board, local government and/or directors support the implementation of digital solutions (variable STR_3);
	STR_4	The most important thing in my work is to ensure a good patient experience (variable STR_4).
Competency e-health readiness	KOMP_1	We use tools and methods related to patient’s experience, such as personas and travel maps, to design and modify digital solutions (variable KOMP_1);
	KOMP_2	We use digital tools to promote innovation, collaboration and mobility for physicians, healthcare professionals and administrations (variable KOMP_2);
	KOMP_3	We have competent leaders (supervisors) for everyday implementation of digital solutions (variable KOMP_3);
	KOMP_4	We invest in targeted training and digital education in all areas and at all levels of our organization (variable KOMP_4);
	KOMP_5	The specialists serving our critical digital solutions are best in class (variable KOMP_5);
	KOMP_6	Employees in our organization have developed digital competences (variable KOMP_6).
Cultural e-health readiness	KUL_1	We have clear and measurable goals to measure the success of our digital solutions implementations (variable KUL_1);
	KUL_2	Each employee understands how their tasks are related to the effectiveness of digital solutions implementation (variable KUL_2);
	KUL_3	We have measures oriented towards patient satisfaction survey (e.g., Net Promoter Score) (variable KUL_3);
	KUL_4	We investigate how the channels of contact with the patient (e.g., visit, teleportation) work together to ensure continuity of the patient care process (variable KUL_4);
	KUL_5	For us, conclusions from research and patient relations have a real impact on the selection and verification of digital solutions (variable KUL_5);
	KUL_6	We use the conclusions of research and patient relations in the experimentation, design, and development of digital solutions (variable KUL_6);
	KUL_7	We systematically draw conclusions from the operation of digital solutions and improve them (variable KUL_7);
	KUL_8	We clearly communicate our digital vision both internally and externally (variable KUL_8);
	KUL_9	We accept the risk to enable experimentation and innovation initiative among employees (variable KUL_9);
	KUL_10	We work with partners and suppliers to create better solutions for our patients (variable KUL_10).
Structural e-health readiness	ORG_1	In our organization, the priority is the continuity of the patient care process and not focusing on the individual tasks of individual employees (variable ORG_1);
	ORG_2	We have defined, described and repeatable processes for the implementation of digital solutions (variable ORG_2);
	ORG_3	We dedicate appropriate resources to work on digitization (variable ORG_3);
	ORG_4	Our organizational model encourages collaboration between doctors, medical and administrative staff and IT specialists (variable ORG_4);
	ORG_5	Medical, administrative, and technological employees jointly develop a plan for the implementation of digital solutions (variable ORG_5);
	ORG_6	New ideas, solutions, or improvements in the organization of tele-consultancy came mainly from the management of the facility (variable ORG_6);
	ORG_7	New ideas, solutions, or improvements in the organization of tele-consultancy came mainly from the employees of the facility (variable ORG_7).
Technological e-health readiness	TECH_1	We have a digital budget that is flexible and allows you to change priorities (variable TECH_1);
	TECH_2	We have a flexible, iterative, and collaborative approach to developing digital solutions (variable TECH_2);
	TECH_3	We use modern architectures (API, cloud, etc.) to promote the speed and flexibility of implementing digital solutions (variable TECH_3);
	TECH_4	We regularly use emerging technologies (e.g., voice interfaces, augmented reality, artificial intelligence, blockchain, etc.) to improve the patient care process (variable TECH_4);
	TECH_5	When providing IT support, we focus on the continuity of the patient care process, not only on the availability of IT systems (variable TECH_5).

**Table 3:** Measures of the constructs’ quality in the model.

Construct	Composite Reliability	Average Variance Extracted (AVE)
KOMP	0.892	0.624
KUL	0.886	0.566
ORG	0.885	0.720
STR	0.814	0.594
TECH	0.848	0.583

**Table 4:** The Fornell–Larcker criterion for discriminant validity.

	KOMP	KUL	ORG	STR	TECH
KOMP	0.790				
KUL	0.716	0.752			
ORG	0.766	0.744	0.849		
STR	0.686	0.664	0.712	0.771	
TECH	0.734	0.728	0.666	0.631	0.763

**Table 5:** Variance Inflation Factor.

	KOMP	KUL	ORG	STR	TECH
KOMP					2.734
KUL			1.000		2.532
ORG					2.984
STR	1.000	1.000			
TECH					

**Table 6:** Path coefficients and significance of relations between constructs.

Hypothesis	Regression Path	Path Coefficients	*p* Values	Interpretation: Hypothesis
H.1	STR → KOMP	0.686	0.000	Confirmed (α = 1%)
H.2	STR → KUL	0.664	0.000	Confirmed (α = 1%)
H.3	KOMP → TECH	0.403	0.000	Confirmed (α = 1%)
H.4	KUL → TECH	0.388	0.000	Confirmed (α = 1%)
H.5	ORG → TECH	0.069	0.279	Not confirmed
H.6	KUL → ORG	0.744	0.000	Confirmed (α = 1%)

## Data Availability

Data are contained within the article.
